# Development of a vesicular stomatitis virus pseudotyped with herpes B virus glycoproteins and its application in a neutralizing antibody detection assay

**DOI:** 10.1128/mbio.01092-24

**Published:** 2024-06-07

**Authors:** Hitomi Kinoshita, Souichi Yamada, Takuma Ogawa, Phu Hoang Anh Nguyen, Shizuko Harada, Madoka Kawahara, Keita Ishijima, Ken Maeda, Hideki Ebihara, Shuetsu Fukushi

**Affiliations:** 1 Department of Virology 1, National Institute of Infectious Diseases, Tokyo, Japan; 2 Department of Veterinary Science, National Institute of Infectious Diseases, Tokyo, Japan; Columbia University Medical College, New York, New York, USA

**Keywords:** herpes B virus (BV), herpes simplex virus (HSV), glycoproteins, pseudotypes, vesicular stomatitis virus (VSV), neutralization assay

## Abstract

Herpes B virus (BV) is a zoonotic virus and belongs to the genus *Simplexvius*, the same genus as human herpes simplex virus (HSV). BV typically establishes asymptomatic infection in its natural hosts, macaque monkeys. However, in humans, BV infection causes serious neurological diseases and death. As such, BV research can only be conducted in a high containment level facility (i.e., biosafety level [BSL] 4), and the mechanisms of BV entry have not been fully elucidated. In this study, we generated a pseudotyped vesicular stomatitis virus (VSV) expressing BV glycoproteins using G-complemented VSV∆G system, which we named VSV/BVpv. We found that four BV glycoproteins (i.e., gB, gD, gH, and gL) were required for the production of a high-titer VSV/BVpv. Moreover, VSV/BVpv cell entry was dependent on the binding of gD to its cellular receptor nectin-1. Pretreatment of Vero cells with endosomal acidification inhibitors did not affect the VSV/BVpv infection. The result indicated that VSV/BVpv entry occurred by direct fusion with the plasma membrane of Vero cells and suggested that the entry pathway was similar to that of native HSV. Furthermore, we developed a VSV/BVpv-based chemiluminescence reduction neutralization test (CRNT), which detected the neutralization antibodies against BV in macaque plasma samples with high sensitivity and specificity. Crucially, the VSV/BVpv generated in this study can be used under BSL-2 condition to study the initial entry process through gD–nectin-1 interaction and the direct fusion of BV with the plasma membrane of Vero cells.

**IMPORTANCE:**

Herpes B virus (BV) is a highly pathogenic zoonotic virus against humans. BV belongs to the genus *Simplexvius*, the same genus as human herpes simplex virus (HSV). By contrast to HSV, cell entry mechanisms of BV are not fully understood. The research procedures to manipulate infectious BV should be conducted in biosafety level (BSL)-4 facilities. As pseudotyped viruses provide a safe viral entry model because of their inability to produce infectious progeny virus, we tried to generate a pseudotyped vesicular stomatitis virus bearing BV glycoproteins (VSV/BVpv) by modification of expression constructs of BV glycoproteins, and successfully obtained VSV/BVpv with a high titer. This study has provided novel information for constructing VSV/BVpv and its usefulness to study BV infection.

## INTRODUCTION

Herpes B virus (BV), also known as macacine alphaherpesvirus 1, belongs to the *Simplexvirus* genus of the *Alphaherpesvirinae* subfamily and *Orthoherpesviridae* family. The genomic structure and antigenicity of BV are similar to those of human herpes simplex virus (HSV) type 1 (HSV-1) and type 2 (HSV-2), which belong to the same genus (1). Macaque monkeys, including rhesus macaque (*Macaca mulatta*), cynomolgus macaque (*Macaca fascicularis*), and Japanese macaque (*Macaca fuscata*), are the natural hosts of BV. Macaque monkeys are also used as laboratory animals in biomedical research to study human disease. In macaque monkeys, BV infection is usually asymptomatic or causes very mild disease, whereby the virus establishes a latent infection in the sensory nerve ganglia and can be reactivated; this process is similar to that of HSV infection in humans (2, 3). However, in a non-natural host species, BV infection is highly pathogenic. In humans, BV infection is severe, resulting in permanent neurological deficit or death. Without timely and adequate intervention measures, case fatality rates can reach 80% (4). Human BV infections are rare, with only 50–60 cases documented globally since the first reported case of human BV infection in 1933 (5). In 2019, two human BV infection cases were identified for the first time in Japan; the presence of infection was confirmed by detecting BV genome sequences in the cerebrospinal fluid (CSF) of patients (6).

Herpesviruses are large, enveloped, double-stranded DNA viruses. HSV encodes over 84 viral proteins; at least 12 of these are envelope glycoproteins, some of which are involved in viral entry (7). Unlike many other enveloped viruses, which only require one or two viral glycoproteins to mediate receptor binding and fusion, HSV requires four viral glycoproteins (gB, gD, gH, and gL) for cell entry (8–11). In the general model of herpesvirus entry into the cell, the receptor-binding protein gD first binds to its cell surface receptor(s). This interaction activates a heterodimeric complex composed of gH and gL (gH/gL), which induces a conformational change in the viral fusion protein gB (10, 12, 13). gB, gH, and gL, which comprise the core fusion machinery in the herpesvirus family, are conserved entry glycoproteins. However, the receptor-binding glycoproteins are diverse and not conserved among the herpesvirus subfamilies (13–15). Nectin-1, also called herpesvirus entry mediator C (HevC), is a cell–cell adhesion molecule and a major cell surface receptor for gD of BV. Meanwhile, four receptors have been identified for gD of HSV-1: nectin-1, nectin-2, herpesvirus entry mediator (HVEM), and modified heparan sulfate (16). The amino acid sequence identity of gB between BV and HSV-1/-2 is approximately 80%, whereas gD and gH/gL are less conserved, ranging from 53% to 66% (17). Although not essential for viral entry, gC is also involved in the attachment of HSV-1 to heparan sulfate proteoglycans on the cell surface (18, 19). By contrast to that of HSV-1, the entry mechanism of BV has not been fully elucidated.

Recently, the production of vesicular stomatitis virus (VSV) pseudotyped with HSV-1 entry glycoproteins was reported (20). The study demonstrated that the four glycoproteins (gB, gD, gH, and gL) of HSV-1 were not only necessary but also sufficient for HSV-1 cell entry. However, the cell entry mechanism of this VSV-pseudotyped virus might not be the same as that of native HSV-1, because it infected neither Vero nor HeLa cells expressing the gD receptor nectin-1, both of which have been widely used in HSV-1 infection research (21).

Because BV is highly pathogenic to humans, it should be studied in biosafety level (BSL)-4 facilities (22). By contrast, VSV-pseudotyped viruses can be handled at a standard BSL-2 facility. Taking advantage of its lower risk to humans, VSV pseudotyped with BV glycoproteins (VSV/BVpv) is a useful tool for the elucidation of the complex cell entry mechanism of BV. In this study, we successfully generated a novel VSV/BVpv, which infected Vero, a cell line highly susceptible to BV infection (23, 24). By modifying the signal peptide domain of BV glycoproteins using an expression vector, which permitted the cell surface expression of BV glycoproteins, we obtained a high-titer VSV/BVpv preparation. Using this VSV/BVpv, we then developed a pseudotype-based neutralizing antibody assay to detect anti-BV antibodies in macaque plasma.

## MATERIALS AND METHODS

### Macaque plasma samples

Plasma samples from 88 macaques (78 from rhesus and 10 from Japanese macaques; 44 females and 44 males), which were captured in Chiba Prefecture in 2019, were used in this study. Of these, plasma sample no. 91 exhibited a high reactivity against BV gH in the immunofluorescence assay (IFA) and was therefore used as a positive BV control antibody in Western blotting.

### Antibodies

The following antibodies were used in this study: anti-hemagglutinin (HA; clone 16B12; BioLegend, San Diego, CA, USA), anti-nectin-1 (clone R1.302; EXBIO, Vestec, Czech Republic), anti-VSV-M-protein (clone 23H12; Merck, Darmstadt, Germany), anti-HSV-1+HSV-2-gB (clone 10B7; Abcam, Cambridge, UK), rabbit anti-VSV-G-protein (described previously [25]), Alexa-Fluor-488-conjugated anti-mouse IgG (A-11001; Thermo Fisher Scientific, Waltham, MA, USA), horseradish peroxidase (HRP)-conjugated anti-mouse IgG (62-6520; Thermo Fisher Scientific), and anti-human IgG (A18811; Thermo Fisher Scientific).

### Cell culture

African green monkey kidney (Vero), baby hamster kidney (BHK)-21, human embryonic kidney (293T), Chinese hamster ovary (CHO)-K1, and human neuroblastoma (IMR-32) cell lines were obtained from the American Type Culture Collection (Manassas, VA, USA). Vero cells were maintained in Dulbecco’s modified Eagle medium (DMEM; Fujifilm Wako Chemicals, Osaka, Japan) supplemented with 5% heat-inactivated fetal bovine serum (FBS; Cytiva, Marlborough, MA, USA) and 1% penicillin–streptomycin (Merck). BHK-21 and 293T cells were maintained in DMEM supplemented with 10% FBS and 1% penicillin–streptomycin. CHO-K1 cells were maintained in Ham’s F-12 nutrient mix (Thermo Fisher Scientific) supplemented with 10% FBS and 1% penicillin–streptomycin. IMR-32 cells were maintained in Eaglet’s minimum essential medium (EMEM; Fujifilm Wako Chemicals) supplemented with 10% FBS, 1% non-essential amino acid solution (Thermo Fisher Scientific), and 1% penicillin–streptomycin. Insect Sf9 cells and Tn5 cells, derived from *Spodoptera frugiperda* and *Trichoplusia ni*, respectively, are used to construct BV glycoprotein-expressing recombinant baculovirus and for the production of recombinant BV glycoprotein, respectively. Sf-9 cells were maintained in Sf-900 II SFM (Thermo Fisher Scientific) supplemented with 10% FBS and kanamycin sulfate (Thermo Fisher Scientific). Tn5 cells were maintained in TC-100 insect medium (Thermo Fisher Scientific) supplemented with 10% FBS, 2% tryptose phosphate broth (BD Difco, Franklin Lakes, NJ, USA), and kanamycin sulfate. Mammalian cell lines were cultured at 37°C in 5% CO_2_ and the insect cell lines at 26°C.

### Plasmids used for the generation of pseudotyped viruses

Genetic sequences encoding the BV glycoproteins (gB, gD, gH, gL, and gC) were amplified from total DNA extracted from the CSF of BV-infected patients (6) by PCR using the KOD FX Neo polymerase (TOYOBO, Osaka, Japan), and their sequences were subsequently determined. Human-codon-optimized DNA sequences encoding the BV gH, gL, and gC glycoproteins were then synthesized (Integrated DNA Technologies, Coralville, IA, USA) to improve the efficiency of cloning into the expression vector. DNA sequences corresponding to the 28-, 24-, 37-, 24-, and 31-amino-acid (signal peptide region) truncations at the N-terminus for BV gB, gD, gH, gL, and gC, respectively (Fig. S1), were subcloned into the pDisplay mammalian expression vector (Thermo Fisher Scientific) at the *Sfi* I and *Sal* I sites using the In-Fusion HD cloning kit (Takara Bio, Shiga, Japan). In these constructs, the sequences corresponding to the vector’s signal peptide (Ig κ-chain leader), which precedes the HA-tag, were connected to each N-terminus-truncated glycoprotein. The DNA sequences of these constructs were verified by Sanger sequencing.

### Production of VSV pseudoviruses

Pseudotype VSVs bearing BV glycoproteins were generated as follows. Briefly, 293T cells (approximately 1 × 10^5^ cells/well of a 12-well plate) were grown on collagen-I-coated tissue culture plates (Corning, Corning, NY, USA) and were co-transfected with a combination of expression plasmids encoding each BV glycoprotein (200 ng each/well of a 12-well plate) using the *Trans*IT-LT1 transfection reagent (Mirus Bio LLC, Madison, WI, USA). At 48 h after transfection, the cells were washed with phosphate-buffered saline (PBS) once and inoculated with G-complemented (*G) VSV∆G-encoding luciferase as reporter gene (designated here as VSVpv) at a multiplicity of infection (MOI) of 0.2 (26). After 1.5 h of incubation at 37°C, the cells were washed with PBS three times and were resuspended in 5% FBS-DMEM. After 24 h of incubation, the supernatants (containing VSV pseudoviruses) were centrifuged to remove cell debris and were stored at −80°C until use. VSV pseudoviruses expressing gB, gD, gH, and gL were called VSV∆G-BDHL; those expressing gB, gH, and gL were called VSV∆G-BHL; and those expressing gB, gD, gH, gL, and gC were called VSV∆G-BDHLC. Mock virus was prepared from 293T cells transfected with the empty pDisplay vector (800 or 1,000 ng/well of a 12-well plate). An equal volume (5 µL) of VSV pseudoviruses produced in parallel was used as an inoculum in the infection experiments to assess the role of gD or gC on infectivity. Briefly, Vero cells (approximately 5 × 10^4^ cells) seeded in 96-well plates were infected with 5 µL of VSV pseudoviruses. At 24 h after infection, the infectivity of the pseudoviruses was assessed by measuring the luciferase activity using the Bright-Glo luciferase assay system (Promega, Madison, WI, USA) and GloMax Discover microplate reader (Promega); infectivity was expressed in relative light units (RLUs). To confirm the incorporation of BV glycoproteins into VSV particles, VSVpv or VSV∆G-BDHL (designated as VSV/BVpv) were purified by ultracentrifugation in 25% sucrose solution. The viral proteins were then subjected to SDS-PAGE and Western blotting (see later section).

### Production of polyclonal antibodies against BV glycoproteins

To obtain antisera against the BV glycoproteins, recombinant proteins were produced using a baculovirus expression system. Briefly, the coding sequences for the soluble form of gD (sgD_1–341_) and the full length of gL (due to the absence of the transmembrane region) were tagged with the 8× His-tag sequence at the 3′ end and were cloned into the baculovirus transfer vector pAcYM1 via the *Bam*HI site (27). The transmembrane topology of the glycoproteins was predicted using TMHMM Server v2.0 (http://www.cbs.dtu.dk/services/TMHMM/). Recombinant baculoviruses were then generated by co-transfection of each transfer vector and the BestBac linearized baculovirus DNA (Expression Systems) into the Sf-9 cells using the FuGENE HD transfection reagent (Promega). Recombinant baculoviruses expressing sgD_1–341_ and gL were labeled as AcYM1-BV-sgD_1–341_ and AcYM1-BV-gL, respectively. Recombinant BV-sgD_1–341_ was prepared from Tn5 cells infected with AcYM1-BV-sgD_1-341_. The culture supernatant of Tn5 cells inoculated with AcYM1-BV-sgD_1–341_ was dialyzed with PBS and was purified on a column packed with Ni-NTA agarose (QIAGEN, Venlo, The Netherlands). The recombinant BV-gL was purified from the lysates of Tn5 cells inoculated with AcYM1-BV-gL, also by Ni-NTA agarose column purification. Polyclonal antibodies against BV-gD and BV-gL were generated in BALB/c mice by immunizing the animals with purified recombinant proteins (10 µg injected four times), which were administered alongside the TiterMAX Gold adjuvant (Merck).

### Cloning and expression of recombinant nectin-1

The nectin-1-coding gene (*HVEC*, GenBank: AF060231) was amplified by RT-PCR from RNA extracted from HeLa cells and cloned into the pKS336 mammalian expression vector (28).

### Treatment of Vero cells with endosomal acidification inhibitors

To examine the effect of treatment of endosomal acidification inhibitors on VSV/BVpv entry, Vero cells were pretreated with bafilomycin A1 (Merck), ammonium chloride (Fujifilm Wako Chemicals), and chloroquine diphosphate salt (Merck). Bafilomycin A1 was prepared in dimethyl sulfoxide (DMSO) to make a stock solution (20 µM). Ammonium chloride and chloroquine were diluted in deionized distilled water to make 1.0 M and 20 mM stock solutions, respectively. The Vero cells were pretreated with increasing concentrations of these inhibitors for 1 h at 37°C, and then the cells were infected with VSVpv or VSV/BVpv (equivalent to ca. 2–5 × 10^5^ RLU/well). Infectivity of pseudoviruses was determined as described in “Production of VSV pseudoviruses.”

### Detection of BV neutralizing antibodies in macaque plasma samples

For the pseudotype-based chemiluminescence reduction neutralization test (CRNT), heat-inactivated (56°C, 30 min) macaque plasma samples or medium only (no-plasma control) were diluted with 2% FBS-DMEM and were mixed with an equal volume of VSV/BVpv (equivalent to ca. 1 × 10^6^ RLU/well); the final dilution was 1:20 (plasma to medium). After a 1-h incubation at 37°C, the mixture was inoculated into Vero cells seeded on 96-well plates (Porvair Sciences, King’s Lynn, Norfolk, UK); the luciferase activity was measured 24 h later. Neutralizing antibody activity was measured as a decrease in chemiluminescence relative to the no-plasma control (relative percent infectivity). The plaque reduction neutralization test (PRNT) was performed using HSV-1 strain F. Serially diluted (fourfold, starting from 1:5 dilution) macaque plasma samples (100 µL per sample) were mixed with an equal volume of HSV-1 (100 PFU/100 µL) and were incubated for 1 h at 37°C. Then, 100 µL of the mixture was inoculated into duplicate wells of Vero cells seeded in 24-well culture plates. After a 1-h incubation, the inoculum was removed and replaced with fresh medium containing γ-globulin (vol/vol, 100:2) (Merck). After 2 d, the cells were fixed with 10% formalin and were stained with crystal violet solution. Finally, the number of plaques was counted. The PRNT (i.e., the HSV-1 NT) titer was calculated based on the reciprocal of the plasma dilution needed to achieve a ≥50% reduction in plaque count (PRNT_50_).

### Immunofluorescence assay

Each of the BV glycoprotein expression vectors or the empty vector (250 ng/well of an 8-well chamber slide) was transfected into 293T cells seeded on collagen-coated plates. After a 48- or 72-h incubation, the transfected cells were fixed with 10% formalin in PBS. The cells were treated with a primary anti-HA antibody for 1 h at 37°C and then with a secondary Alexa-Fluor-488-conjugated anti-mouse IgG for 1 h at 37°C. The cells were examined on Vectashield mounting medium containing 4′,6-diamidino-2-phenylindole (DAPI) (Vector Laboratories, Newark, CA, USA) under a fluorescence microscope (BZ-X800; Keyence). To verify the detection of nectin-1, CHO-K1 cells, which are naturally nectin-1 deficient (29), were transfected with a plasmid encoding nectin-1 or an empty vector. Expression of nectin-1 was confirmed by IFA using an anti-nectin-1 primary antibody and a secondary Alexa-Fluor-488-conjugated anti-mouse IgG.

### Western blotting

To detect the viral proteins within the VSV particles, the purified viral particles were subjected to SDS-PAGE and Western blotting. Briefly, 293T cells, transfected with expression plasmids encoding the BV glycoproteins, were lysed in PBS containing 1% NP40. The lysates were then mixed with SDS sample buffer solution (Fujifilm Wako Chemicals) and were boiled to denature the proteins. The proteins were fractioned by SDS-PAGE on 12.5% or 5%–20% e-PAGELs (ATTO, Tokyo, Japan). The protein bands were stained using Bio-Safe Coomassie Premixed Staining Solution (Bio-Rad, Hercules, CA, USA) or were transferred to a polyvinylidene fluoride (PVDF) membrane (Merck). The membranes were blocked with PBS containing 0.05% Tween-20 (PBST) and 5% skim milk, and were washed with PBST before being stained with primary antibodies against viral proteins (i.e., VSV G and M proteins; HSV-1 and HSV-2 gB; and BV gD, gL, and HA), the HA-tag, or macaque plasma (BV-antibody-positive control). Finally, an HRP-conjugated secondary antibody was applied to the membranes, and the bands were visualized using the chemiluminescent detection reagent (Cytiva) and ImageQuant LAS 500 chemiluminescent imager (GE HealthCare).

### Statistical analysis

Data analysis and visualization were performed using GraphPad Prism 8 (GraphPad software, San Diego, CA, USA). An unpaired *t*-test with Welch’s correction was used to determine statistically significant differences between groups. The diagnostic efficiency of the CRNT was determined initially by constructing receiver operating characteristic (ROC) and two-graph ROC curves using StatFlex ver.7 (Artech, Osaka, Japan). The cutoff value (COV) was determined using the Youden index. The relationship between the HSV-1-based PRNT_50_ and the VSV/BVpv-based CRNT results (relative percent infectivity) was evaluated using Spearman’s rank correlation coefficient. The threshold for statistical significance was defined as a *P* value < 0.05.

## RESULTS

### Expression of BV glycoproteins

**Fig 1:**
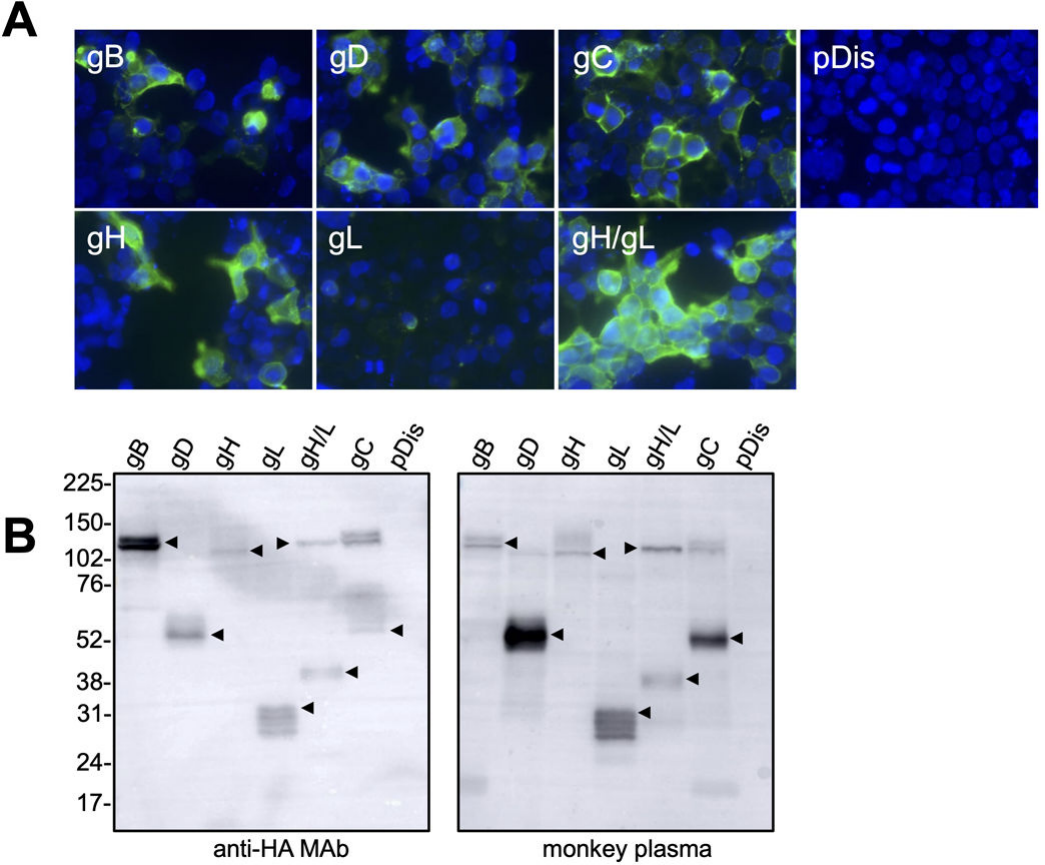
Expression of B virus glycoproteins. (**A**) 293T cells were transfected with plasmids encoding BV glycoproteins. After a 48– to 72-h incubation, the cells were fixed with 10% formalin and were stained with an anti-HA monoclonal antibody detected with a secondary Alexa-Fluor-488-conjugated antibody. (**B**) The plasmid-transfected cells were also analyzed by Western blotting using an anti-HA monoclonal antibody and anti-BV-antibody-positive macaque plasma. Molecular weight marker labels (kDa) are shown on the left, and the protein of interest in each lane is indicated by an arrow.

The four glycoproteins (gB, gD, gH, and gL) of HSV-1 play essential roles in viral entry (13). In addition to these glycoproteins, gC increases the infectivity of VSV pseudotypes harboring HSV-1 glycoproteins (21). Here, we constructed mammalian expression plasmids encoding each of the five BV glycoproteins (gB, gD, gH, gL, and gC). These glycoproteins contained the N-terminus cell surface targeting signal instead of the native signal peptide sequences, which was followed by the HA epitope tag. The expression plasmids were transfected into 293T cells, and protein expression was examined by IFA using an anti-HA-tag antibody (Fig. 1A). The expression of gB, gD, gC, and gH was clearly observed, and they were localized on the cell surface and within the cytoplasm. In contrast, only a faint staining of gL was observed (Fig. 1A). However, when gH and gL were co-transfected, the cell surface and cytoplasm expression of the gH/gL complex was clearly visible. In addition, the molecular sizes of gH and gL observed in the co-transfected cells were higher than those detected in cells expressing either protein alone (Fig. 1B). This result is consistent with a previous report demonstrating that the HSV-1 gL acts as a chaperone for gH and is involved in the transport of gH to the cell membrane (30, 31). Western blotting confirmed that the expressed gB, gD, and gC had the desired molecular weights (Fig. 1B).

### Generation of VSV pseudotyped with BV glycoproteins

**Fig 2:**
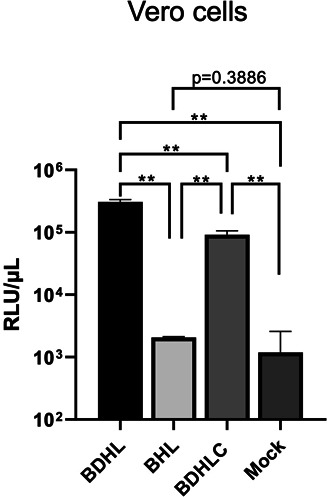
Infectivity of VSV pseudoviruses. The indicated combinations of plasmids expressing BV glycoproteins were co-transfected into 293T cells. After infecting 293T cells with VSV∆G/Luc-*G, VSV pseudoviruses expressing gB, gD, gH, and gL (VSV∆G/Luc-BDHL); gB, gH, and gL (VSV∆G/Luc-BHL); or gB, gD, gH, gL, and gC (VSV∆G/Luc-BDHLC) were produced. Mock virus was prepared from 293T cells transfected with the empty vector. Each pseudovirus was used to infect Vero cells seeded into 96-well plates. After a 24-h incubation, the luciferase activity of each well was measured using a luminometer. Data are presented as the mean of three experiments with standard deviation (SD). Significance was conducted using a two-tailed Student’s *t*-test with Welch’s correction. **P < 0.01.

To generate VSV pseudotyped with BV glycoproteins, various combinations of the expression plasmids encoding the BV glycoproteins described above were co-transfected into 293T cells. Upon inoculation of the seed virus encoding luciferase as reporter gene (VSVpv) into 293T cells, VSV pseudoviruses were collected from the cell culture supernatants and their infectivities were measured using the luciferase assay. We did not determine the viral titer quantitatively nor measure the viral protein amount of pseudoviruses, which is often required to standardize the inoculum (32). Instead, we performed the infection experiments using equal volumes of the supernatants containing pseudoviruses that were prepared in parallel to overcome this limitation. The VSV∆G/Luc-BDHL, which contained gB, gD, gH, and gL, exhibited the highest level of luciferase activity. Meanwhile, VSV∆G/Luc-BHL, which contained gB, gH, and gL, but not gD, had a relatively low luciferase activity, which was almost the same as that of the negative control VSV pseudovirus (Fig. 2). However, VSV∆G/Luc-BDHLC, which expressed gB, gD, gH, gL, and gC, exhibited lower infectivity rates than VSV∆G/Luc-BDHL. Similar results were obtained when the VSV seed pseudovirus expressing a GFP reporter (VSV∆G/GFP-*G) was used to produce VSV pseudoviruses (Fig. S2). Collectively, these results showed that BV infectivity was highest when the four glycoproteins gB, gD, gH, and gL were used to generate the VSV pseudovirus (i.e., VSV∆G/Luc-BDHL). The infectivity level of VSV∆G/Luc-BDHL was two-log higher than the background level (Fig. 2) and was similar to that of VSV pseudoviruses bearing viral glycoproteins reported in the previous studies (33, 34). It was noted that gB expression was highest among glycoproteins (Fig. 1B). No additional examinations were performed to assess whether the altered expression of gB (or other glycoproteins) affects VSV pseudovirus infectivity, because the VSV∆G/Luc-BDHL infectivity seemed to be sufficient for further experiments to examine viral entry as well as to measure neutralizing antibody. Therefore, we decided to use this VSV pseudovirus bearing BV glycoproteins BDHL expressing luciferase (designated as VSV/BVpv) in subsequent experiments.

### Incorporation of BV glycoproteins into VSV virions

**Fig 3:**
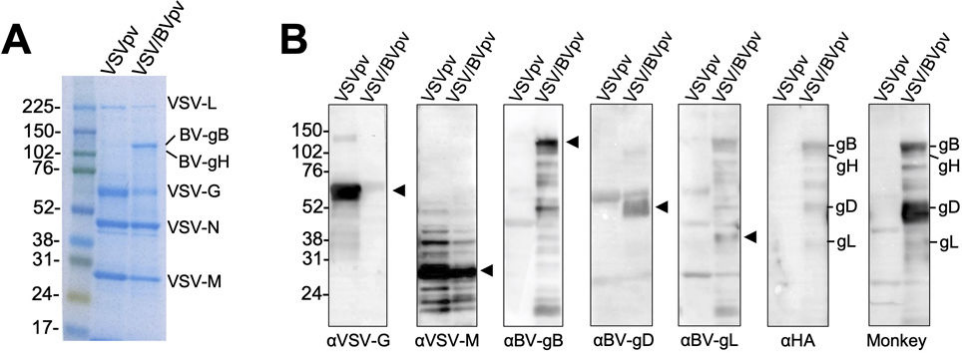
Incorporation of BV glycoproteins into VSV pseudotype particles. Pseudotyped VSV∆G/Luc bearing the BV glycoproteins gB, gD, gH, and gL (VSV/BVpv), and VSV∆G/Luc-*G (VSVpv) were partially purified by ultracentrifugation in 25% sucrose medium, separated by SDS-PAGE, and analyzed by Coomassie blue staining (**A**) or by Western blotting (**B**) using the indicated antibodies. Monkey, BV-positive macaque plasma. Molecular weight marker labels (kDa) are shown on the left, and the proteins of interest in each lane are indicated by an arrow or a label.

To confirm the successful incorporation of BV glycoproteins into VSV particles, VSV/BVpv and its seed virus VSVpv were purified by ultracentrifugation and were subjected to SDS-PAGE. Protein bands corresponding to the VSV proteins L (241 kDa), N (47 kDa), and M (27 kDa) were detected in both the VSV/BVpv and the VSVpv virions (Fig. 3A). Protein bands corresponding to the VSV protein G (63 kDa) and the BV gB (ca. 120 kDa) were only detected in the VSVpv or VSV/BVpv preparations, respectively. The four BV glycoproteins (gB, gD, gH, and gL) were detected in the VSV/BVpv virions by Western blotting (Fig. 3B). These results indicate that BV glycoproteins were successfully incorporated into the VSV pseudovirus particle.

### Infection of cells with VSV/BVpv

**Fig 4:**
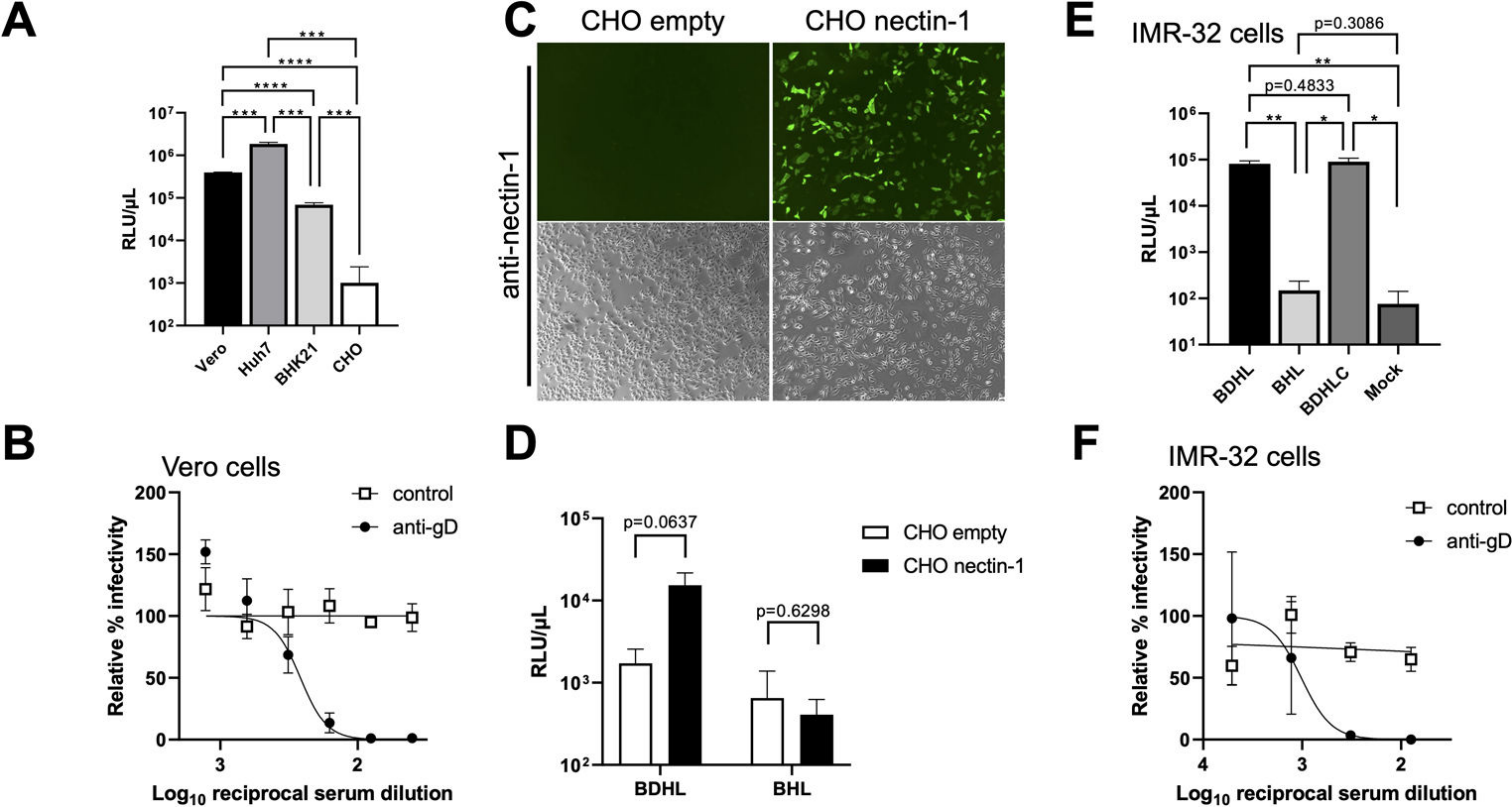
Entry of VSV/BVpv into Vero cells is dependent on gD and its receptor nectin-1. (**A**) The VSV/BVpv bearing the four entry essential glycoproteins gB, gD, gH, and gL was used to infect various cell lines. Briefly, 50 µL of the 10-fold-diluted VSV/BVpv was used to infect the Vero, Huh7, BHK-21, and CHO-K1 cell lines seeded into 96-well plates. After a 24-h incubation, the luciferase activity was measured. (**B**) The VSV/BVpv was preincubated with serially diluted anti-gD mouse serum or control serum. The mixture was then inoculated into Vero cells. The relative infectivity of VSV/BVpv mixed with the serum samples versus that of the no-serum control is shown. (**C**) CHO-K1 cells were transfected with a nectin-1 expression plasmid or an empty vector. The expression of nectin-1 on CHO-K1 cells detected by an anti-nectin-1 monoclonal antibody is shown (top images: fluorescence field; bottom images: bright field). (**D**) CHO-K1 cells, transfected with a nectin-1 expression plasmid or an empty vector, were subsequently infected with VSV pseudovirus bearing the four entry essential glycoproteins gB, gD, gH, and gL (VSV∆G/Luc-BDHL), or that lacking gD (VSV∆G/Luc-BHL). The infectivity of each pseudovirus determined by measuring luciferase activity is shown. (**E**) VSV∆G/Luc-BDHL, VSV∆G/Luc-BHL, and VSV∆G/Luc-BDHLC, which expressed gB, gD, gH, gL, and gC, were infected to IMR-32 cells, and the luciferase activity was measured at 24 h post-infection. (**F**) VSV∆G/Luc-BDHL was preincubated with diluted anti-gD mouse serum or control serum and then inoculated into IMR-32 cells. The luciferase activity was determined as described in panel **B**. Data are represented as the mean of three experiments with standard deviation (SD). Significance was conducted using a two-tailed Student’s *t*-test with Welch’s correction. *P < 0.05, **P < 0.01, ***P < 0.001, ****P < 0.0001.

The infectivity of VSV/BVpv was examined by using various cell lines. Huh7 cells were highly susceptible to VSV/BVpv infection, followed by Vero and BHK-21 cells. However, CHO-K1 cells showed no susceptibility to the VSV/BVpv infection (Fig. 4A).

BV cell entry is primarily mediated by the interaction between gD and its receptors nectin-1 or nectin-2 on the cell surface. Of note, nectin-1 is more effective than nectin-2 at mediating BV cell entry (35). To investigate whether the infection of VSV pseudovirus generated in this study was dependent on gD, VSV/BVpv was inoculated into the naturally nectin-1-expressing Vero cells (21) in the presence of increasing concentrations of anti-gD or negative control antibodies. The infectivity of VSV/BVpv was blocked by the anti-gD antibody but not by the negative control antibody in a dose-dependent manner (Fig. 4B). Next, CHO-K1 cells, which do not naturally express nectin-1 and are therefore not BV permissive (29), were used to examine the nectin-1 dependency of VSV/BVpv. When CHO-K1 cells transfected with empty vector were inoculated with VSV/BVpv (“CHO empty” in Fig. 4C), only the background level of luciferase activity was observed (Fig. 4D), which was comparable to the luciferase activity of CHO-K1 cells inoculated with the gD-deficient pseudovirus (“BHL” in Fig. 4D). By contrast, when VSV/BVpv was inoculated into CHO-K1 cells expressing nectin-1 (“CHO nectin-1” in Fig. 4C), the luciferase activity was 8.9-fold higher than that of the CHO empty inoculated with the VSV/BVpv (Fig. 4D). These results indicate that VSV/BVpv infection was dependent on the presence of gD and nectin-1. We next investigated the infectivity of VSV/BVpv in human neuroblastoma-derived cells. When IMR-32 cells were infected with VSV∆G/Luc-BDHL (BDHL; expressing gB, gD, gH, and gL), a high level of luciferase activity (approximately 10^5^ RLU/μL) was observed, while VSV∆G/Luc-BHL (BHL; lacking gD) showed a little infectivity (Fig. 4E). Consistent with the experiments using Vero cells, the enhancement of the infectivity by adding gC expression in preparation of VSV/BVpv was not observed (VSV∆G/Luc-BDHLC, Fig. 4E), and the infection of VSV/BVpv to IMR-32 cells was inhibited by anti-gD antibody (Fig. 4F).

### Effect of endosomal acidification inhibitors on the cell entry of VSV/BVpv

**Fig 5:**
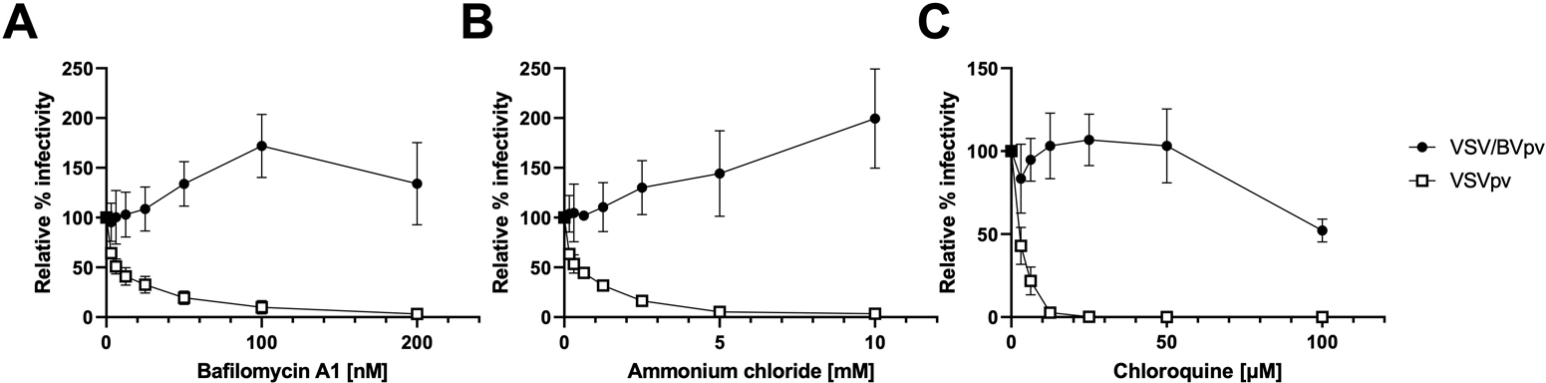
Entry of VSV/BVpv into Vero cells is not affected by inhibitors of endosomal acidification. Vero cells were pretreated with increasing concentrations of bafilomycinA1 (**A**), ammonium chloride (**B**), or chloroquine (**C**) for 1 h, and then VSV/BVpv or VSVpv was added to the cells in the presence of inhibitors. After a 24-h incubation, the infectivity was determined by measuring luciferase activity. Data are presented as the mean of three experiments with standard deviation (SD).

It has been shown that an exposure of endosomal acidification inhibitors on Vero cells does not inhibit HSV-1 infection, indicating that HSV-1 can fuse directly with the plasma membrane when infected to Vero cells, but not internalized through low-pH-dependent endocytic pathway (36). To examine the entry pathway of the VSV/BVpv in Vero cells, Vero cells were pretreated with endosomal acidification inhibitors, such as bafilomycin A1, ammonium chloride, or chloroquine, and then the cells were inoculated with VSV pseudoviruses. As shown in Figure 5, infection of seed virus VSVpv to Vero cells was inhibited by treatment with all reagents tested in a dose-dependent manner, consistent with the fact that VSVpv utilize endocytosis and a low-pH-dependent fusion (37). In contrast, endosomal acidification inhibitors did not inhibit VSV/BVpv entry. These results indicated that VSV/BVpv enters to Vero cells via the pH-independent direct fusion pathway, as shown in the entry study of HSV-1 (36).

### Detection of BV neutralizing antibodies using macaque samples

**Fig 6:**
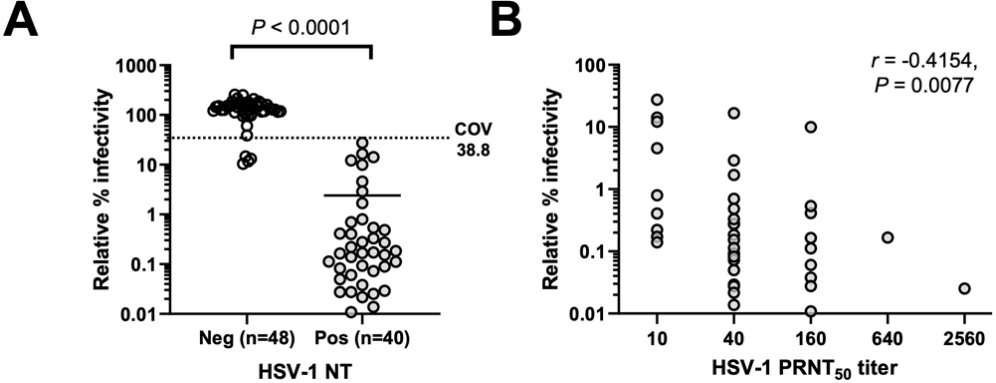
Neutralization assays with macaque plasma samples. (**A**) Correlation of neutralization assay results of HSV-1-based PRNT with the relative infectivity determined using VSV/BVpv-based CRNT. The HSV-1-based PRNT and VSV/BVpv-based CRNT were performed using plasma samples (*n* = 88) obtained from macaques. The COV of the relative infectivity of VSV/BVpv is shown as a dotted line. The significance (*P* < 0.0001) of the difference between the positive and negative samples of HSV-1-based PRNT is also shown. Bars represent the mean of relative infectivity of VSV/BVpv of each group. (**B**) Comparison of relative infectivity determined using the VSV/BVpv-based CRNT and the neutralizing antibody titer determined using the HSV-1-based 50% PRNT (PRNT_50_) (*n* = 40). The Spearman’s rank correlation coefficient was −0.4154 (95% confidence interval: −0.6492 to −0.1099, *P* = 0.0077).

We next tested the performance of the generated VSV/BVpv in a CRNT to detect neutralizing antibodies against BV in macaque plasma. The detection accuracy of the CRNT was compared against that of the PRNT, which uses HSV-1. Because of its cross-reactivity against BV antigens and less pathogenic nature, HSV-1 has been conventionally used to detect antibodies against BV in monkeys (38–41). Among the 88 macaque plasma samples collected for this study, 48 were negative and 40 were positive for HSV-1 in the PRNT. The same macaque plasma samples were used to determine the relative percent infectivities of VSV/BVpv in the CRNT. The mean relative percent infectivity of HSV-1-positive plasma samples (2.4%, range: 0.01%–27.4%) was significantly lower than that of the HSV-1-negative samples (134.5%, range: 10.4%–254.4%) in the PRNT (*P* < 0.0001) (Fig. 6A). Among the 40 HSV-1-positive samples in the PRNT, the relative percent infectivity of each sample was moderately negatively correlated with the HSV-1 PRNT_50_ titer (correlation coefficient: −0.4154, *P* < 0.01) (Fig. 6B). The ROC and two-graph ROC curves were used to evaluate the accuracy of the CRNT in detecting neutralizing antibodies against BV (Fig. S3). The COV of the relative percent infectivity on the CRNT was determined as 38.8%. Plasma samples that had a relative percent infectivity less than the COV were considered as BV-antibody-positive in the CRNT. The sensitivity, specificity, positive predictive value, and negative predictive value of the CRNT using VSV/BVpv, which were calculated using the HSV-1 PRNT as the gold standard, were 100% (40/40), 91.7% (44/48), 90.9% (40/44), and 100% (44/44), respectively (Table 1). Four specimens that were negative in the HSV-1 PRNT were classified as positive in the CRNT; their relative percent infectivities were 10.4%, 11.7%, 13.2%, and 14.6%.

**TABLE 1:** Comparing the performance of the conventional and new neutralization tests using macaque plasma samples^*a*^

VSV/BVpv CRNT	HSV-1 PRNT		
	Positive	Negative	Total
Positive	40	4	44
Negative	0	44	44
Total	40	48	88

^*a*^ PRNT, plaque reduction neutralization test using HSV-1; CRNT, VSV-pseudotype-based chemiluminescence reduction neutralization test using VSV/BVpv.

## DISCUSSION

We successfully generated VSV/BVpv bearing the four BV glycoproteins gB, gD, gH, and gL, as well as a luciferase reporter. VSV/BVpv efficiently infected the Vero cell line, which has been widely used to study BV cell entry. The properties of VSV/BV/Luc were similar to those of infectious BV with regard to gD and its cellular receptor nectin-1 being required for cell entry. This was evidenced by the fact that VSV/BVpv infection of BV-permissive Vero cells was inhibited in the presence of an anti-gD antibody, while VSV/BVpv infection of the BV-nonpermissive CHO-K1 cells was increased on expression of nectin-1. We showed that VSV/BVpv could infect not only Vero cells and nectin-1-expressing CHO-K1 cells, but also various other cell lines, such as Huh7 (human hepatoma), BHK-21 (baby hamster kidney), and IMR-32 (human neuroblastoma) cells. The requirement of gD during the infection of IMR-32 cells with VSV/BVpv was also confirmed, indicating that the infection of human neural cells with BV was gD dependent (Fig. 4). A particular merit of this study was that we were able to generate VSV/BVpv with a high degree of infectivity (>10^5^ RLU/μL) upon inoculation of Vero cells, which could be used directly in neutralization assays. By contrast, VSV pseudotype viruses bearing other viral envelope proteins sometimes require additional concentration steps (e.g., via ultracentrifugation), which are laborious and time-consuming (20).

The pCAGGS mammalian expression vector has been previously used for the expression of viral glycoproteins to generate many VSV pseudotype viruses (34, 42, 43). However, when we generated VSV/BVpv in cells transfected with plasmids constructed based on the pCAGGS vector, and inoculated it into Vero cells, minimal luciferase activity was observed (data not shown). By contrast, when we used the pDisplay mammalian expression vector to express BV glycoproteins, in which a native signal sequence of each glycoprotein was replaced with Ig κ-chain leader sequence, to generate VSV/BVpv, a much higher level of Vero cell infectivity was achieved. A high-level infectivity of VSV pseudovirus bearing HSV-1 glycoproteins on Vero cells was also observed when pDisplay vector was used for the expression of HSV-1 glycoproteins (gB, gD, gH, and gL) (Fig. S4). The discrepancy between these results obtained from the use of different expression vectors might be related to the different properties of VSV and BV glycoproteins implicated in the viral maturation process in transfected cells. The VSV virion is coated with an envelope G glycoprotein. After synthesis and glycosylation in the Golgi apparatus, the G protein is transported to the plasma membrane, where it is incorporated into the VSV virion during the budding process (44). Herpesviruses, however, use a different mechanism, whereby the outer envelope, which is studded with viral glycoproteins, is acquired in the Golgi or the *trans*-Golgi network; these enveloped virions are then transported to the plasma membrane in vesicles (45, 46). Therefore, expressing BV glycoproteins with native viral signal peptides may have prevented them from being transported to the plasma membrane for efficient incorporation into the VSV pseudotype particles. However, further research is needed to confirm this theory, for example, by conducting a detailed analysis of the intracellular distribution and/or transport kinetics of BV glycoproteins connected to naïve or heterologous leader sequences.

Hilterbrand et al. (21) have shown the enhancement by gC in a VSV system bearing HSV-1 glycoproteins. It should be noted that the enhancement is restricted in the specific cell types (CHO-HVEM and HaCaT cells) (21). In contrast, no enhancement by gC was observed for VSV pseudovirus bearing BV glycoproteins infected to IMR-32 cells (Fig. 4E), nor for VSV pseudovirus bearing HSV-1 glycoproteins infected to Vero cells (Fig. S4). Furthermore, a reduction of VSV pseudoviruses bearing BV glycoproteins by gC was observed when using the Vero cells (Fig. 2). This discrepancy probably comes from different cell types used. Consistent with these findings, it has been shown that, by using gC-deficient recombinant HSV-1, gC supports an HSV-1 entry to CHO-HVEM and HEKa cells during endosomal low-pH pathway but not to Vero and IMR-32 cells (47). We suggested that BV entry process varied by cell types with regard to the requirement of gC.

Comparison of the relative infectivity of VSV/BVpv in the CRNT with that of HSV-1 in the PRNT showed that the CRNT was more sensitive than the PRNT. Using the CRNT, four HSV-1-negative samples were reclassified as being BV- positive (relative infectivity range: 10.4%–14.6%) (Fig. 6A).

There are limitations in this study. First, we were not able to perform any experiments with the infectious BV because BV is not available for research purposes at present in Japan. Instead, we used the gold standard HSV-1 PRNT as a surrogate BV neutralization assay to determine the presence of anti-BV antibodies in macaque plasma samples. A previous study reported that neutralizing antibodies in monkey sera generally neutralize both BV and HSV-1 (38). However, the higher sensitivity of CRNT (using VSV/BVpv) as compared with that of PRNT (using HSV-1) may be explained by the fact that neutralizing antibodies against BV in macaque plasma may not fully react to HSV-1 neutralizing epitopes. In general, reporter-based assays are simpler, faster, and more sensitive than traditional serological assays. Thus, the results of this study suggest that the CRNT using VSV/BVpv is a useful tool for determining the presence of neutralizing antibodies against BV in plasma samples.

Another limitation of this study is that VSV pseudoviruses do not reflect the native lipid/glycoprotein composition of BV particles. Therefore, VSV pseudoviruses do not necessarily imply the same infection properties and/or antigenicity of native BV. However, like a native BV, entry of VSV/BVpv was dependent on gD and nectin-1 (Fig. 4). In addition, there was no inhibitory effects of VSV/BVpv infection on Vero cells treated with endosomal acidification inhibitors (Fig. 5). This entry process was consistent with infection of native HSV-1 to Vero cells (36). Therefore, VSV/BVpv would be useful to understand an initial entry process through gD–nectin-1 interaction and a direct fusion of BV glycoproteins with the plasma membrane of Vero cells.

In conclusion, we have developed a VSV pseudovirus bearing the four essential glycoproteins of BV. We produced high titers of VSV/BVpv without performing additional concentration steps and showed that it infected a variety of cell types using the same cell entry route as BV. Furthermore, we developed a VSV-pseudotype-based CRNT system to detect the presence of anti-BV neutralizing antibodies in plasma samples. We demonstrated that the CRNT was simple, rapid, highly sensitive, safe, and did not require a BSL-4 facility. However, due to the cross-antigenicity between BV and HSV-1, the CRNT may not be effective at specifically diagnosing BV infection in humans; thus, the test must be further improved to differentiate between the anti-BV and anti-HSV-1 antibody response. Meanwhile, the VSV/BVpv pseudotype system might be useful in investigating anti-BV antibody in monkey plasma samples.

## Data Availability

Genetic sequences encoding the BV gB, gD, gH, gL, and gC obtained in this study are available at Genbank database: accession numbers LC785695, LC785697, LC785698, LC785699, and LC785696 for gB, gD, gH, gL, and gC, respectively.

## SUPPLEMENTAL MATERIAL

Figure S1 mbio.01092-24-s0001.tif Construction of the BV glycoprotein expression plasmids.

Figure S2 mbio.01092-24-s0002.tif Production of GFP-VSV pseudoviruses.

Figure S3 mbio.01092-24-s0003.tif ROC and two-graph ROC analyses.

Figure S4 mbio.01092-24-s0004.tif Infectivity of VSV pseudoviruses bearing HSV-1 glycoproteins.

ASM does not own the copyrights to Supplemental Material that may be linked to, or accessed through, an article. The authors have granted ASM a non-exclusive, world-wide license to publish the Supplemental Material files. Please contact the corresponding author directly for reuse.
