# Supplementary figures and images for: Development of a vesicular stomatitis virus pseudotyped with herpes B virus glycoproteins and its application in a neutralizing antibody detection assay
Source: mBio. 2024 Jun 7;15(7):e01092-24. doi: 10.1128/mbio.01092-24 (PMC11253632; doi:10.1128/mbio.01092-24)

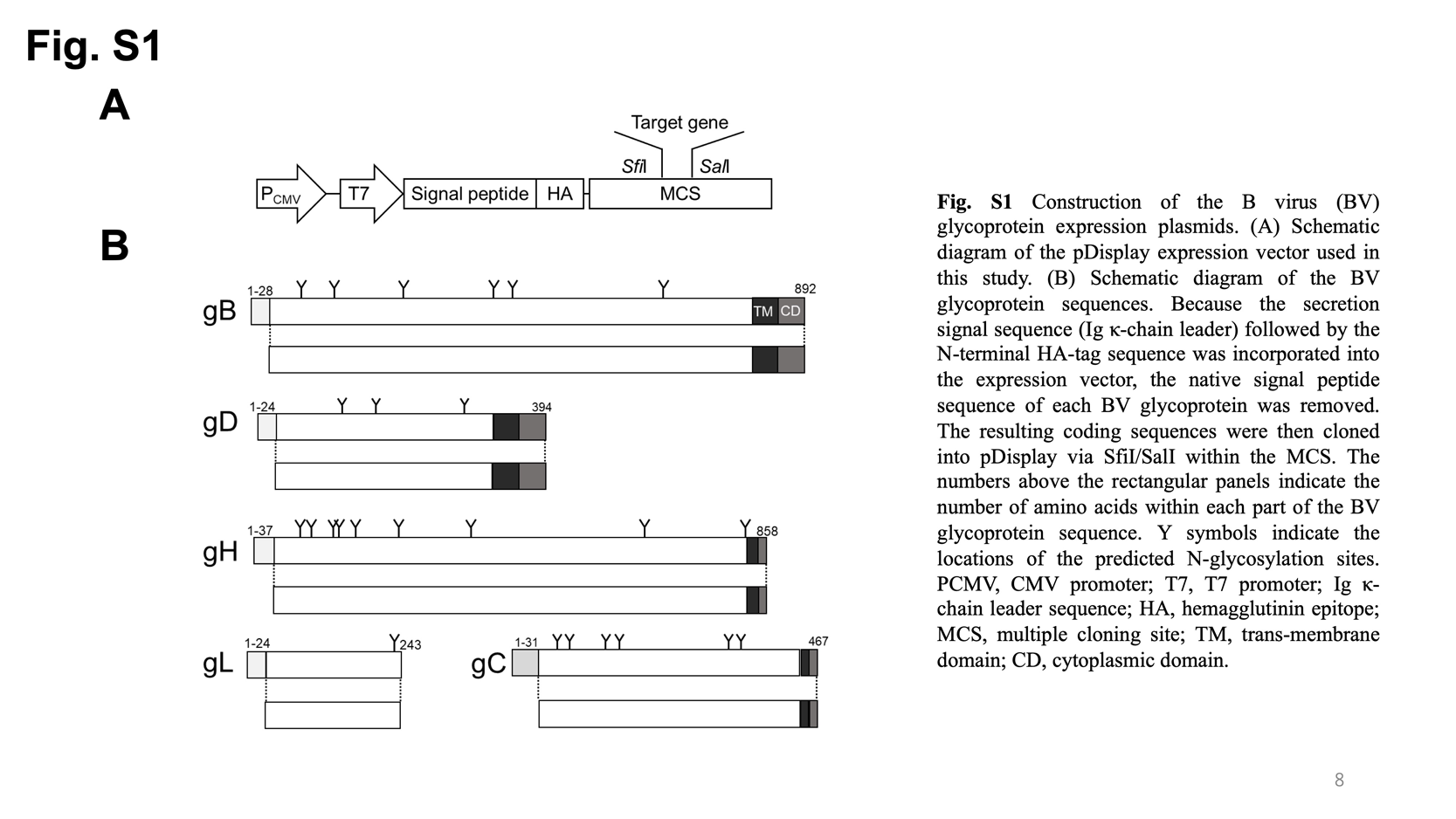

Supplement: Figure S1 — Construction of the BV glycoprotein expression plasmids. [file mbio.01092-24-s0001.tif]

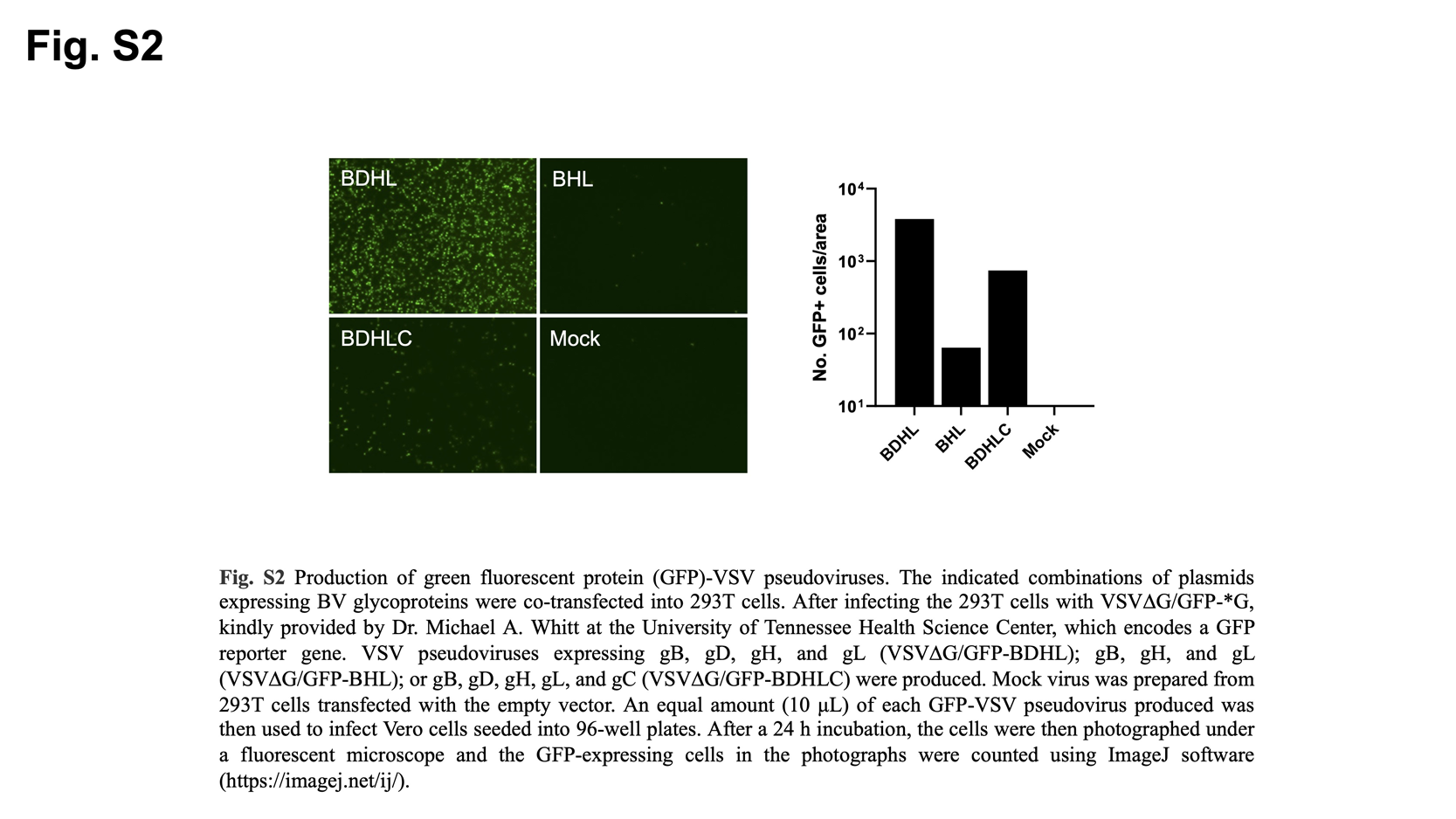

Supplement: Figure S2 — Production of GFP-VSV pseudoviruses. [file mbio.01092-24-s0002.tif]

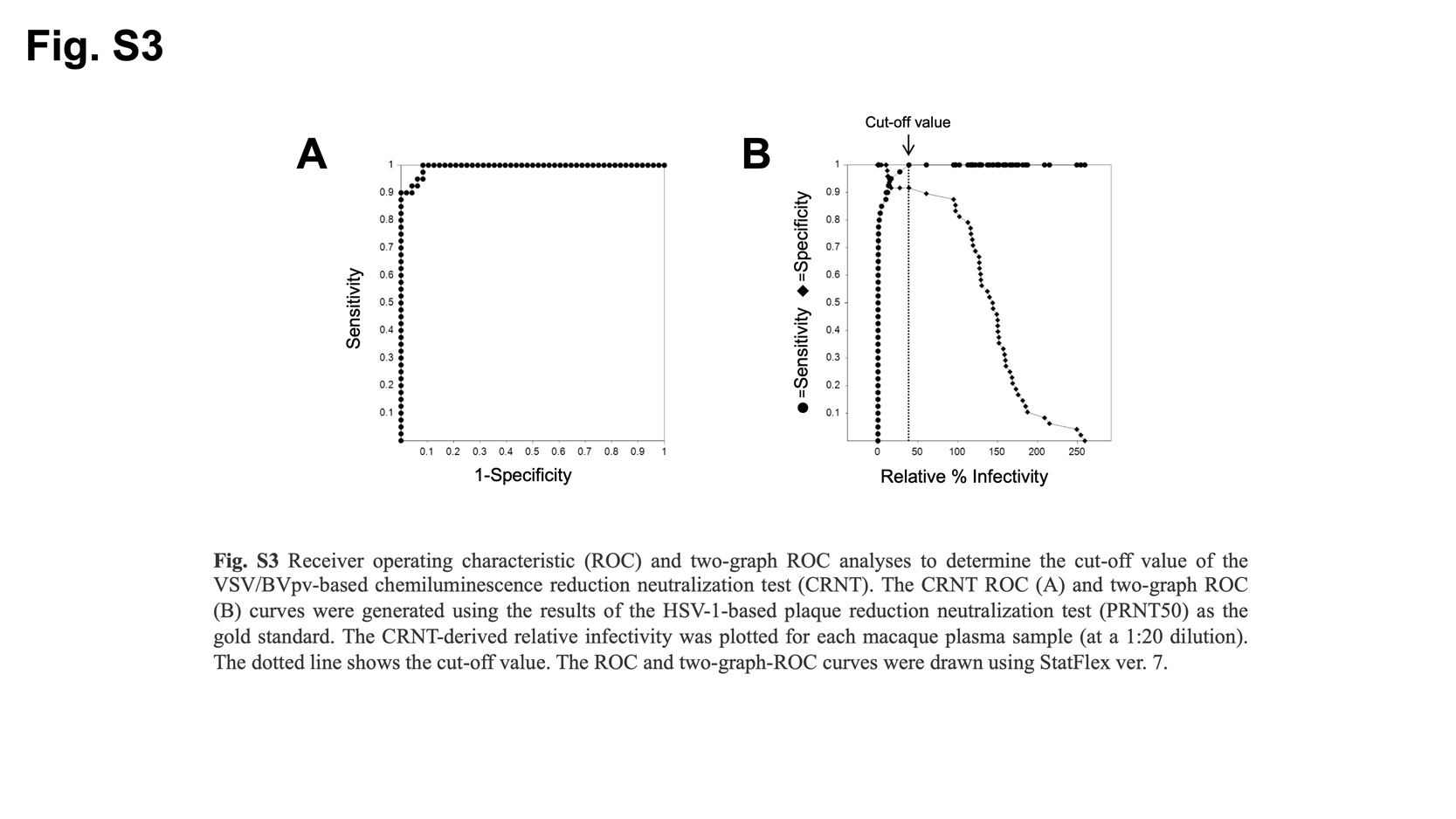

Supplement: Figure S3 — ROC and two-graph ROC analyses. [file mbio.01092-24-s0003.tif]

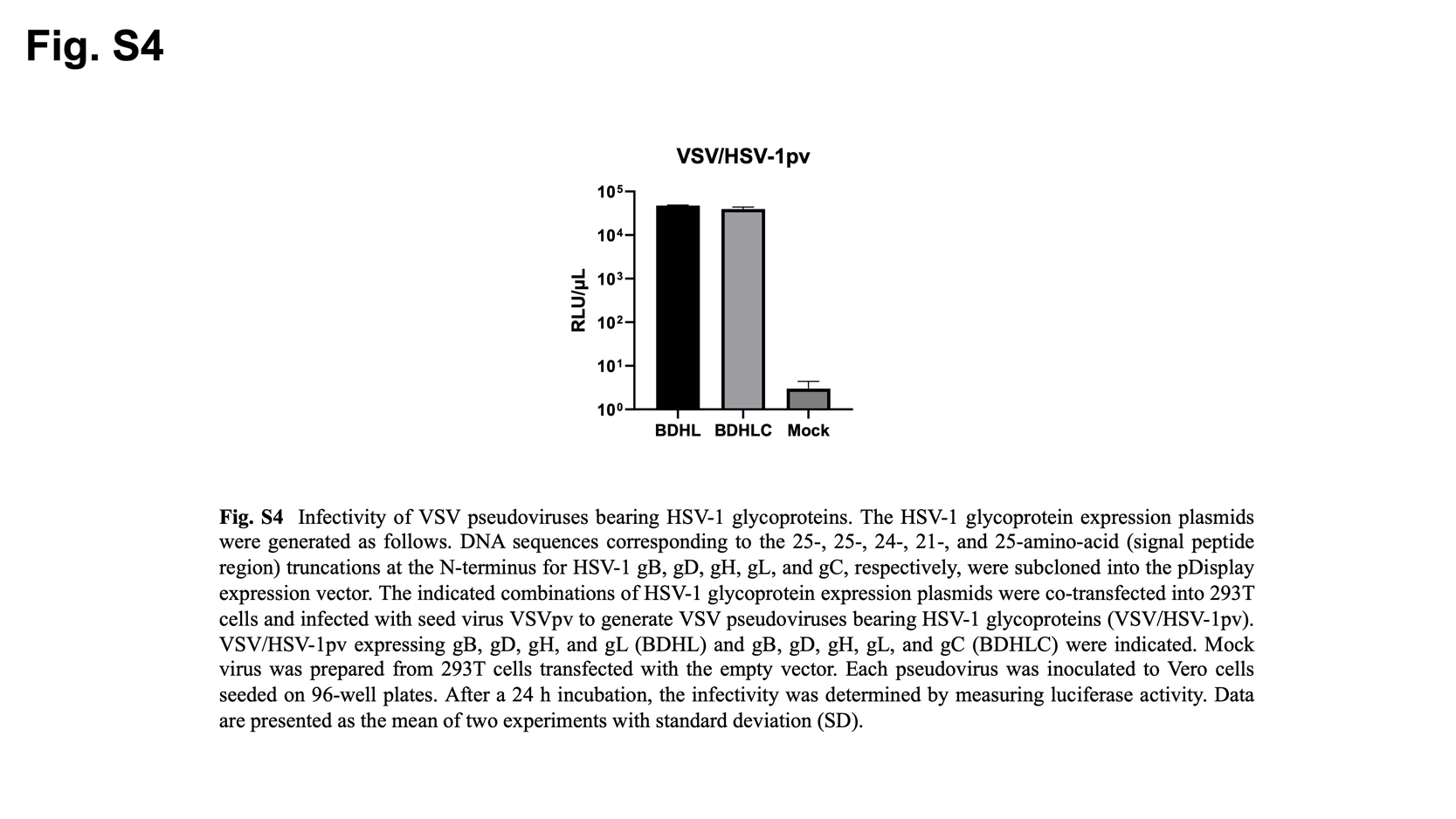

Supplement: Figure S4 — Infectivity of VSV pseudoviruses bearing HSV-1 glycoproteins. [file mbio.01092-24-s0004.tif]
